# Visual evoked potential in the early diagnosis of glaucoma. Literature review

**Published:** 2020

**Authors:** Anne Marie Firan, Sînziana Istrate, Raluca Iancu, Ruxandra Tudosescu, Radu Ciuluvică, Liliana Voinea

**Affiliations:** * Barnsley District Hospital, NHS Trust, Barnsley, South Yorkshire, Great Britain; ** Ophthalmology Department, University Emergency Hospital Bucharest, Bucharest, Romania; *** Ophthalmology Department, Regina Maria - Health Private Care, Bucharest, Romania; **** Anatomy Department, “Carol Davila” University of Medicine and Pharmacy, Bucharest, Romania

**Keywords:** glaucoma, visual evoked potentials, latency, optic nerve

## Abstract

Visual evoked potentials (VEP) are a significant visual electrophysiological diagnostic exam, which can be used as a suitable objective measure of optic nerve function. The topic was greatly debated and many correlations between the magnitude of the VEP latency parameters and parameters of Humphrey static perimetry suggested that the abnormal cortex responses in patients with glaucomatous changes could be tested by both electrophysiological and physical methods. Moreover, the optic nerve glaucomatous damage observed by reduction in ganglion cell layer and retinal fibre layer thickness through SD-OCT tests, which are known to precede Humphrey visual field defects, correlates with VEP latency parameters, thus consolidating the position of the VEP testing in glaucoma progression.

## Introduction

Even today, glaucoma is considered an eye condition that can progress to major vision loss and blindness. Owing to its insidious onset and lack of symptoms, patients frequently come late at ophthalmology check-up. In some instances, optic nerve damage continues to progress despite adequate treatment and good intraocular pressure (IOP) control. According to data from the World Health Organization, the prevalence of glaucoma for the population of 40–80 years old is 3.54%. Prevalence rates of primary open angle glaucoma (POAG) was found higher in African population (4.20%), while the prevalence of primary angle closure glaucoma (PACG) is highest in the Asian population (1.09%). In 2013, the number of people (ages between 40–80 years) who had glaucoma was estimated to be 64.3 million, numbers that are going to increase to 76.0 million in 2020 and the forecast by 2040 is to become close to 111.8 million [**1**].

Diagnosis of glaucoma is based on the IOP values, optic nerve head appearance (cupping of optic nerve) in fundus examination, visual field assessment (VFA) for typical glaucomatous field defects, gonioscopy and pachymetry, with invaluable data also from SD-OCT regarding retinal fiber layer thickness (RNFL) and ganglion cell layer thickness (GCC). 

Electrophysiology in glaucoma brings valued information through pattern ERG, which detects macular ganglion cell dysfunction and VEP for nerve fiber layer loss and can be of aid in the evaluation of “glaucoma suspects” with risk factors for glaucoma such as high levels IOP or optic nerve head changes, even before detectable loss trough visual field exam [**2**]. One advantage of the electrophysiological methods is the possibility of testing various processing stages of the visual paths separately, thus electroretinogram (ERG) reflecting the activity of different retinal layers while visual evoked potentials (VEP) represent a brain response to visual stimuli [**3**].

**Fig. 1:**
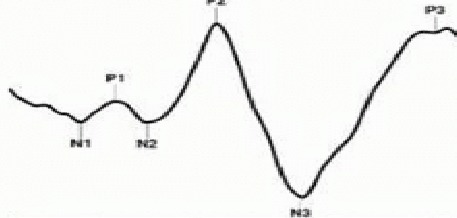
Flash VEP: an initial negative peak (N1) followed by a positive peak; second negative (N2) and second positive (P2) peaks follow, and so on

By measuring the change of electrical signal over the occipital cortex in response to light stimuli, a visual evoked potential (VEP) can be recorded. Studies have shown that abnormal VEPs correlate with a degree of optic nerve damage [**4**,**5**]. To be able to register VEPs, the EEG signal is augmented by repetitive stimulation and light-evoked changes are isolated from the basal EEG reading. A waveform has been identified in normal individuals, characterized by 2 positive and 2 negative waves that alternate. The VEP waveform typically contains a primary negative wave (N1), then a positive wave P1, also known as P100 for its usual location at 100 msec); second negative (N2) and second positive (P2) peaks follow (**Fig. 1**). 

**Fig. 2:**
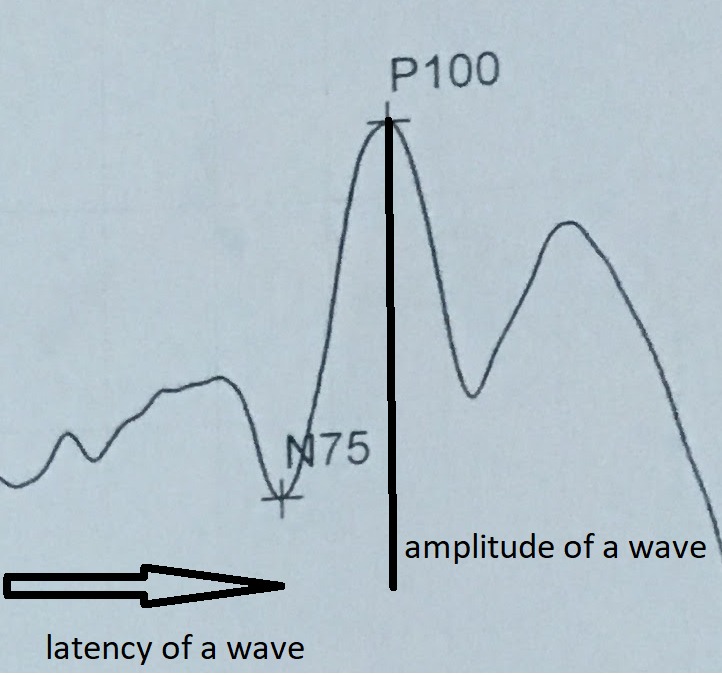
Normal amplitude and latency of a wave in pattern VEP

The amplitude of onset of each peak is measured. Delay in the onset of a peak is referred to as latency and reflects some level of injury of the visual pathway (**Fig. 2**).

The standard protocols used worldwide are:

1) Pattern-reversal VEPs; those are elicited by both large 1-degree (°) checkerboard stimuli as well as small 0.25° checks. 

2) Pattern onset/ offset VEPs; determined by the same checks as (1). 

3) Flash VEPs, which are determined by a flash (brief luminance increment) that subtends a visual field larger or equal with 20° [**6**]. 

**Fig. 3:**
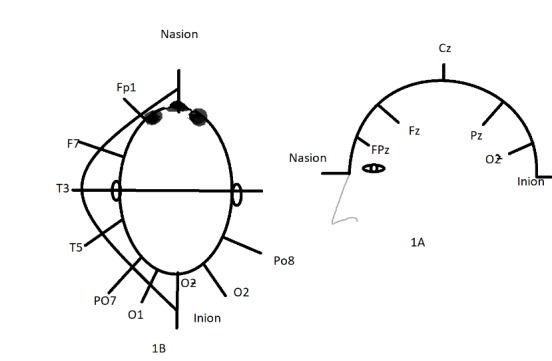
Electrode positions in standard and multi-channel VEP: **1A.** Standard VEP electrodes location. The midline (Oz) electrode is active. The reference electrode is located at location Fz. The midline position is indicated by subscript z. **1B.** Multi-channel VEP active electrodes exam locations PO7, PO8, O1, O2, (lateral electrodes) and Oz (midline electrode location)

The electrodes are placed as follows (**Fig. 3**):

For standard VEP, one electrode is placed on the occipital scalp that corresponds to the visual cortex at O (the active electrode) and another electrode at F (reference electrode). Another electrode is affixed and connected to the ground. Commonly used ground electrode positions include the forehead and vertex (C). Other possibilities are earlobe (A1 or A2), mastoid or linked earlobes (**Fig. 3A**).

International Society for Clinical Electrophysiology of Vision (ISCEV) recommends a minimum of three active electrodes; from those there is one electrode at Oz (midline electrode) while two are placed at O1 and O2 (lateral electrodes), for multi-channel VEP. The active electrodes (one midline and two lateral) should be referenced to Fz. In order to increase sensitivity to lateral asymmetries there is the possibility of placing additional electrodes at PO7 and PO8. Those are referred to Fz as well [**7**]. The positions of the lateral electrodes are illustrated in **Fig. 3B**.

This paper summarized many of the studies that contributed to the impact of the VEP as a measure of optic nerve fiber loss detection in early glaucoma. We also included the papers concerning VEPs utilization in clinical setting for the objective evaluation of visual field defects, especially those that are typical in glaucoma.

**Pattern VEP and flash VEP**

There are two main theories in glaucoma mechanisms; in both the increase of IOP determines destruction of optic nerve fibers which results in the loss of visual function, associated with an alteration of the VEP waveforms. Because of these facts, many studies have followed the presence of a correlation between visual field defects and VEP parameters. So far we acknowledge that the VEP is overshadowed by the foveolar responses and thus reflects macular function, and when the parameters were altered it meant a modification of the central visual field [**8**]. The most used VEP stimulus is full- field pattern-reversal because eyes are evaluated separately; it also focuses on the evaluation of the anterior segment of the visual pathways. For uncooperative patients the most used are flash VEP assesses the continuity of the visual pathways; compared to the than the pattern reversal type, the variability of its latencies has been noted [**9**]. 

A case-control study conducted by Jha et al. measured the VEP as a potential indicator of health status of retinal ganglion cells in POAG. Twenty POAG eyes were followed (group of patients aged 45 to 74 years, all of whom were on beta-blocker drops for IOP control). Another group of 40 individuals between 38 and 72 years old formed the control group, giving a control to case ratio of 2:1. Pattern reversal VEP (PR-VEP) and flash VEP have been assessed by monocular technique, with recordings taken of N75, P100, and N145. They found a significantly prolonged N145 latency in the patient group versus the control with the use of PR-VEP (P=0.011). N75 and P100 latency were prolonged, but not significantly versus the control. The amplitudes of PR-VEP have been significantly reduced in the patient group, while flash VEP had the opposite result of significantly higher amplitudes for the patient group. Compared to the control group, the latencies of flash VEP were not significantly prolonged. In comparing the 2 techniques, flash VEP recorded significantly prolonged latencies compared to PR-VEP. However, PR-VEP was found to elicit significantly higher amplitudes compared to flash VEP, and therefore was more reliable. They concluded that the deterioration in ophthalmic variables caused by POAG was reflected in increased latency and with a difference between the amplitude values of flash VEP versus PR-VEP.

In another case-control study by Watts et al., a decrease of P1’s amplitude was correlated in Humphrey perimetry with the degree of visual field alteration. Flash VEP was used, allowing the analysis of patients with minimal distortion of the results due to opaque media, incorrect refraction, pupillary anomalies, and stimulus presentation. Two groups: first one (POAG group, mean age 66) comprising 74 patients (total 130 eyes) were compared with the second group (control group, mean age 66) of 125 patients (total 250 eyes).The most significant finding was that in the first group, the P1 amplitude was significantly reduced compared to the second group (P=0.001), but the difference was not significant for P2 amplitude measurements. There was also a negative correlation between reduction in P1 and the ratio of cup to disc vertical diameter (P=<0.005), which sustains the potential use of VEP in the evaluation of cases with opaque media where the optic disk is not visible as for 53 patients with unilateral cataract from the second group the difference was not significant in P1 and P2, thus reflecting the flash VEP value in opaque media. Confounding factors, which reduced the P100 amplitude included hypertension, history of cerebrovascular accident and ischaemic heart disease. The 28 POAG patients with evidence of systemic vascular disease had a significantly reduced mean P1 amplitude of 1.74 +- 0.83 uV, while the remaining POAG patients had a mean of 2.92 +- 1.21 uV (P=,0.005). This highlighted the potential for false positive results (reduction of P1) in patients with systemic vascular disease without POAG [**4**].

A further case-control study analyzed the use of PR-VEP in optic nerve functional assessment in early POAG. One hundred twenty subjects in the control group and ninety patients in the POAG group were enrolled in this study that concluded that in the POAG group both P100 and N70 amplitudes were reduced while P100 latency was prolonged [**10**]. Furthermore, Ruchi et al. found that in Humphrey perimetry there was a negative correlation between the MD values of POAG and the P100 latency, parameters that were previous studied and correlated by Horn, Bergua, Jünemann and Korth [**10**,**11**]. According to Towle et al. [**12**], there was a significant correlation between increased pattern VEP latency and Humphrey perimetry defects (the location and severity) , and funduscopic aspect of the optic disc. Parisi et al. also reported that in POAG eyes the latency of P100 was significantly prolonged compared with control group and there was a significant correlation with modifications of the MD [**13**]. In order to record VEPs, other stimuli have been used. A cross-sectional study was conducted using SD-tVEP, which are VEPs with short-duration transient in a group of 84 patients with a mean age of 68 using testing conditions involving high contrast and low contrast testing conditions for 133 eyes with controlled IOP on the examination day. The SD-tVEP values, analyzed using the Diopsy NOVA-LX software, were compared with the parameters of Humphrey perimetry in order to identify eyes with an increased risk of progressive visual field loss. According to HFA 30-2 visual field scores the stage of disease was pre-determined as mild disease, moderate disease, or severe disease. In high contrast testing, the SD-tVEP latency increased in a linear manner with increasing severity of disease (P=0.001). Degree of progression was assessed based on serial visual fields; for each eye the mean was calculated using 12 visual fields or more per eye. The definition of rapid progression was a loss = 0.7 dB/ y. 1/ 3 of all eyes tested demonstrated rapid progression, with 73% of them recording VEP latency abnormalities. In contrast, for the more visually stable eyes, only 47% showed any latency abnormality. Eyes that had a latency abnormality had a disease progression of -0.87 +-0.3dB/ y, whereas those with normal latency had a lower progression rate of -0.32 +-0.4dB/ y. The results showed potential for a single-visit, prognostic test to measure disease progression [**14**]. 

Another study by Greenstein et al. aiming to identify which pathways were damaged by glaucoma concluded that both the magnocellular M- and parvocellular P- pathways were affected. Moreover, several ganglion cell subgroups in the retina can be explored to detect the transmission function of the magnocellular geniculate pathway. Sweep VEP were obtained in both positive and negative luminance contrast being elicited by modulation of an array of 32×32 isolated checks (each check subtending 7.5′ of visual angle) upon a steady “yellow” background. The study groups comprised in 14 control subjects, 15 POAG patients and 7 glaucoma suspects and the iso-luminant chromatic stimulus conditions appeared to favor the P-pathway, whereas the luminance contrast stimuli favored the M-pathway. For patients with POAG the VEP responses were significantly reduced in both pathways. However, the glaucoma suspect group had significantly reduced VEP responses only for the 15-Hz positive luminance contrast condition, which might mean that in early POAG the most affected pathway is the “on”-subdivision of the M-pathway [**15**].

Recently, the isolated-check VEP (icVEP) technique has been reported to detect optic neuropathy due to glaucoma earlier and faster [**16**]. “Isolated-check” refers to a specific cell or pathway that can be assessed. This study enrolled patients in two groups: a POAG group with 25 patients and a control group with 20 healthy patients. The examination of the ganglion cell layer (GCC) was performed by OCT. Results of this study performed by Chen et al. on Neucodia visual electrophysiological diagnostic system (MKWH AMD, Huzhou Medconova Medical Technology, Inc.) suggested that icVEP has a similar diagnostic power to OCT measurements of GCC in early glaucoma detection if the comparison is realized using quantitative data [**17**]. Also, when using the icVEP for early POAG, it detected visual function anomalies in approximately 60% of the eyes with q specificity as high as 90% in a study by Fan et al. For Fan et al., Signal To Noise Ratio (SNR) correlated with both a RNFL thickness decrease and central visual field loss severity [**18**].

**Multifocal VEP**

Multifocal VEP (mfVEP) was determined to be an effective investigation in the assessment of early glaucomatous changes in patients with unreliable or unconfirmed visual field modification. Graham SL et al have followed 436 consecutive cases (retrospective assessment) suggested as glaucoma suspects were followed within a year and mfVEP changes were correlated with disease progression stages and had an overall sensitivity of 97.5% in detecting glaucoma with established subjective field loss. The group with low-risk suspects had normal mfVEP in 92.2%. A subgroup was tested using masked disc assessment alone, and, in this group, mfVEP had a similar sensitivity in detecting anomaly in Humphrey perimetry measurements, but with a higher specificity (89.2% vs. 79.5%) [**3**].

Alterations of implicit times and amplitudes of N2 response in the central area might be superior in early glaucoma detection compared to Humphrey perimetry assessment in a study by Golemez et al. [**19**]. In a study of 126 eyes divided in 4 groups: 30 healthy, 28 glaucoma suspect, 48 early glaucoma and 20 advanced glaucoma cases with investigations performed every 3 months in a 6 months-period results showed that implicit times of all mfERG components were significantly delayed in glaucoma, also both delayed implicit time and reduced amplitude of N2 wave in the central area are effective prognosticators in early POAG diagnosis [**14**,**19**].

## Conclusion

From the literature review, we can conclude that VEP is a valuable visual electrophysiological tool. It is useful for POAG patients for the evaluation of optic nerve defects. Unlike the visual field assessments, VEP has the crucial advantage of been completely objective, because the electrophysiological tests are a more unbiassed examination of the function of the optic pathway. This objectiveness is because cognitive factors (like tiredness, stress and others) or patient’s motor skills (especially for the elder patients) are absent. As the cited studies have shown, VEP modifications are significantly associated with optic nerve and visual field specific alterations, which make VEP method a powerful predictor of early POAG.

Further, the relationship between VEP parameters and visual field MD forces the conclusion that physical methods and electrophysiological methods can be used in a complementary way.

Also, the VEP latencies are significantly associated with MD, which raises the possibility that the retina-visual cortex transfer interruption determines the gravity of glaucomatous damage. 

**Acknowledgements**

All authors have equal contribution to the paper. Authors acknowledge the support of the POCU 2014-2020 “Diagnosticul si terapia bolilor rare sistemice cu afectare oculara – OCURARE”. 
